# MODexplorer: an integrated tool for exploring protein sequence, structure and function relationships

**DOI:** 10.1093/bioinformatics/btt062

**Published:** 2013-02-08

**Authors:** Jan Kosinski, Alessandro Barbato, Anna Tramontano

**Affiliations:** ^1^Department of Physics, ^2^Center for Life Nano Science @Sapienza, Istituto Italiano di Tecnologia and ^3^Istituto Pasteur, Fondazione Cenci Bolognetti, Sapienza University, 00185 Rome, Italy

## Abstract

**Summary:** MODexplorer is an integrated tool aimed at exploring the sequence, structural and functional diversity in protein families useful in homology modeling and in analyzing protein families in general. It takes as input either the sequence or the structure of a protein and provides alignments with its homologs along with a variety of structural and functional annotations through an interactive interface. The annotations include sequence conservation, similarity scores, ligand-, DNA- and RNA-binding sites, secondary structure, disorder, crystallographic structure resolution and quality scores of models implied by the alignments to the homologs of known structure.

MODexplorer can be used to analyze sequence and structural conservation among the structures of similar proteins, to find structures of homologs solved in different conformational state or with different ligands and to transfer functional annotations. Furthermore, if the structure of the query is not known, MODexplorer can be used to select the modeling templates taking all this information into account and to build a comparative model.

**Availability and implementation:** Freely available on the web at http://modorama.biocomputing.it/modexplorer. Website implemented in HTML and JavaScript with all major browsers supported.

**Contact:** anna.tramontano@uniroma1.it

**Supplementary information:** Supplementary data are available at *Bioinformatics* online

## 1 INTRODUCTION

Exploring the sequence, structure and function relationships between a protein and its homologs is a powerful strategy to transfer functional annotations such as, for example, ligand-binding sites and/or to detect the existence of alternative conformational states. Likewise, in homology modeling, a thorough survey of all homologs with known structure often can lead to building much better models than obtained by automatically selecting the closest homolog as a template.

Current tools either provide only a general overview of sequence–structure–function relationships [e.g. GeneSilico Metaserver (Kurowski and Bujnicki, 2003) or MESSA (Cong and Grishin, 2012)] or focus on selected aspects, such as ligand-binding sites [e.g. firestar (Lopez *et al.*, 2011)]. A platform for the in-depth integrated analysis of the sequence, structure and function relationships is still missing. Consequently, the information contained in remote relationships may be missed or exploited only through tedious procedures.

Here we describe MODexplorer, a web server that integrates sequence analysis and structure comparison with functional annotations. It provides a more complete and detailed view of the sequence, structural and functional diversity within and between protein families than existing tools. If the structure of the query protein is not known, it permits to select a template for homology modeling and to automatically build the model.

## 2 TOOL DESCRIPTION

**Fig. 1.**
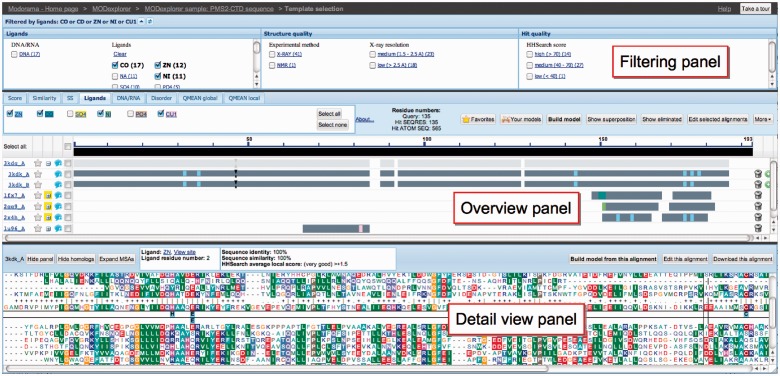
Snapshot of the MODexplorer interface in ‘Ligands’ display mode, where ligand-binding sites are marked on the alignments. The interface is composed of three panels. The filtering panel allows filtering the hits by functional and structural annotations. The overview panel displays the hits as a BLAST-like diagram. The detail view panel enables displaying alignment of the query to currently selected hit along with the MSAs of their families. In this example (query: C-terminal domain of PMS2 protein), users can easily find that one of the structures (3KDK) related to one of the two top-scoring templates (3KDG) contains metal ions associated with conserved motifs (see detail view panel)

A snapshot of the user interface of MODexplorer is shown in Figure 1. A detailed description of integrated software and databases, along with the parameters and references, is included in Supplementary Data and available on the server. MODexplorer accepts as input a protein sequence, PDB code or PDB file. MODexplorer then

- Creates the multiple sequence alignment (MSA) of the query protein family using HHblits (Remmert *et al.*, 2012).
- Generates alignments to proteins of known structure using HHSearch (Söding, 2005) and the PDB database filtered at 70% sequence identity.
- For every HHSearch hit, retrieves and aligns related PDB chains, i.e. chains with a sequence similarity >70%. Such ‘redundant' chains can be useful. For example, they might have been solved in complex with different ligands, represent alternative conformational states or have a better structural quality.
- Displays alignments to the HHSearch hits and their related PDB chains both schematically with a BLAST-like bar diagram and as multiple sequence alignments including query and hit sequences, and their homologs.
- Graphically shows annotations on the alignments. The annotations include ligand- and DNA-/RNA-binding sites, secondary structure (predicted for the query and calculated for chains of known structures), disorder (predicted for the sequence query and estimated by B-factor/missing residue annotations for the structure query and known hit structures), HHSearch similarity scores and QMEAN (Benkert *et al.*, 2011) scores of models built based on the alignments.
- Allows filtering of the hits based on the presence of nucleic acids and other ligands in the structures, on the HHSearch scores, the experimental technique used to solve the structures and the crystallographic resolution.
- Allows modeling of the query based on any selected alignment. Models are built using Modeller (Sali and Blundell, 1993) and evaluated using QMEAN.
- Provides visualization of structural superpositions of PDB chains and model structures in Jmol (http://www.jmol.org/).
- Enables superposition of PDB chains based on pairwise alignment inferred from the alignments to the query. This is useful, for example, to verify if a low scoring hit has a similar structure in the aligned region as a higher scoring one.
- Allows the assessment and modification of the alignments via our interactive alignment editor MODalign (Barbato *et al.*, 2012).
- Provides management utilities such as eliminating the hits and creating a ‘favorites' list of relevant hits.

## 3 CONCLUSIONS

MODexplorer can be used both to explore the protein sequence, structural and functional diversity and to help in template selection in homology modeling. Thanks to tight integration of sequence, structure and function information, MODexplorer provides a comprehensive overview of the features of the target protein and of its homologs, helps in detecting remote homologs and facilitates the selection of templates for modeling.

## Supplementary Material

Supplementary Data
