# Supplementary material for: MODexplorer: an integrated tool for exploring protein sequence, structure and function relationships
Source: Bioinformatics. 2013 Feb 8;29(7):953–4. doi: 10.1093/bioinformatics/btt062 (PMC3605600; doi:10.1093/bioinformatics/btt062)
Supplement: Supplementary Data [file supp_29_7_953__index.html]

MODexplorer: an integrated tool for exploring protein sequence, structure and function relationships — MODexplorer: an integrated tool for exploring protein sequence, structure and function relationships — Supplementary Data 

# MODexplorer: an integrated tool for exploring protein sequence, structure and function relationships

## Supplementary Data

files

**Files in this Data Supplement:**

- Supplementary Data - doc file
