# Supplementary material for: MODexplorer: an integrated tool for exploring protein sequence, structure and function relationships
Source: Bioinformatics. 2013 Feb 8;29(7):953–4. doi: 10.1093/bioinformatics/btt062 (PMC3605600; doi:10.1093/bioinformatics/btt062)
Supplement: Supplementary Data [file supp_btt062_MODexplorer_SUPP_DATA_updated.doc]

# Detailed description of integrated software and databases

## Construction of the query MSA

The MSA of the query protein family is constructed using two iterations of HHblits (Remmert et al., 2012) with default parameters, and using the specially formatted non-redundant UniProt database (The UniProt Consortium, 2011) available from <ftp://toolkit.lmb.uni-muenchen.de/pub/HH-suite/databases/>. HHblits running parameters can be customized using the input form.

## Search for homologs with known structure

Potential homologs with known structure in the PDB (Berman et al., 2003) are searched using HHSearch (Söding, 2005) using default parameters (which include -realign, -mact 0.3, -sc 5, these options can be customized using the input form). The MSA, constructed as described above, is used as a query. HHSearch from the HHSuite version 2.015 is used with a pdb70 (PDB filtered at 70% sequence identity) database downloaded from <ftp://toolkit.lmb.uni-muenchen.de/pub/HH-suite/databases/hhsearch_dbs/>.

## Collecting related PDB chains

For every PDB chain found with HHSearch, the related PDB chains are retrieved based on: 1) lists of PDB chains that were filtered out during database preparation (these lists are included in the records of the HHSearch HMM database) 2) clusters of similar PDB chains from MMDB (Madej et al., 2012) non-redundant PDB chain set (<ftp://ftp.ncbi.nih.gov/mmdb/nrtable/>), created based on all-against-all BLAST comparison and single linkage-clustering with different E-value thresholds (MODexplorer uses the clusters obtained with 10e-80 redundancy threshold). Similar PDB chains derived from both HHSearch and MMDB are merged together and used as a list of related PDB chains in MODexplorer. The use of two sources of similar PDB chains ensures a more complete list of PDB chains highly similar in sequence and overcomes differences in preparation of the databases (e.g. the minimum length of PDB chain).

## Collecting PDB annotations

PDB annotations such as full SEQRES sequences, experimental method, missing residues, crystallographic resolution are retrieved from a local MySQL PDB database using our Python library LocalPDB (<http://biocomputing.it/LocalPDB>) and utilizing DB-LOADER (<http://sw-tools.pdb.org/apps/DB-LOADER/source/source.html>) for creating the database.

## Multiple sequence alignments (MSAs) of hit families

For every HHSearch hit or related PDB chain, the MSAs are obtained from the HHSearch alignment database downloaded weekly from <ftp://toolkit.lmb.uni-muenchen.de/pub/HH-suite/databases/hhsearch_dbs/>. If the MSA is not present in this database for a given PDB chain (since it contains MSAs only for representative PDB chains), the representative PDB chain present in HHSearch database is identified based on clusters of similar PDB chains (see above " Collecting related PDB chains") and the sequence of the given PDB chain is aligned to the MSA of representative PDB chain family using HHAlign from HHSuite.

## Secondary structure and solvent accessibility

Secondary structure and solvent accessibility for the query are predicted using PSI-PRED (Jones, 1999) and ACCpro (Cheng et al., 2005), respectively. The query secondary structure is calculated using DSSP (Kabsch and Sander, 1983) if the query is of known structure. The values for the hits are also computed using DSSP. Secondary structure is displayed as an annotation in the graphical interface. Both secondary structure and solvent accessibility are used for calculating the QMEAN scores (see below).

## Disorder

Disordered regions for the query are predicted using DISOPRED (Ward et al., 2004) using the HHblits MSA as the input. The B-factor values and information about missing residues for the hits are derived from the original PDB records. In the structure input mode, if the PDB code is provided, B-factor values and information about missing residues are retrieved from the PDB record. If the user submits a PDB file, the B-factors are retrieved, if present.

## Ligand and DNA/RNA binding sites

Ligand binding sites are retrieved from a local database of ligand binding sites extracted weekly from the PDBeMotif (Golovin and Henrick, 2008) database. DNA and RNA binding sites are retrieved from a local copy of NPIDB (Spirin et al., 2007). We have selected these databases since they are: 1) regularly updated, 2) comprehensive (e.g. including all chains from PDB and not only the representative ones, 3) downloadable and maintainable as a local copy.

## Sequence similarity annotations

The sequence similarity scores are extracted from the HHSearch output. These values are displayed in MODexplorer in two ways: 1) on the alignment bars as a color gradient and 2) in the detailed alignment panel as a "similarity row", which depicts the similarity between columns as characters (e.g. |, +, . ) in the same way as in the HHSearch output.

## Calculation of QMEAN scores

QMEAN (Benkert et al., 2011) scores are calculated based on models constructed from the HHSearch alignments. Models are built using Modeller (Sali and Blundell, 1993) in 'very_fast' modeling mode and skipping the modeling of the insertions and tails not present in the template. QMEAN is used as implemented in the OpenStructure library (Biasini et al., 2010). For performance reasons, scores for up to 30 HHSearch hits are automatically calculated. Users can calculate QMEAN scores for the remaining hits using the graphical interface.

## Modeling

Models are built using Modeller (Sali and Blundell, 1993) with default options. Within the interface, users can additionally choose the very fast modeling option that skips additional model optimization, skip modeling insertions and tails, and request not to model regions aligned with residues missing in templates that will be treated as chain breaks.

## Structural superpositions

Structure superpositions of models and their templates are performed using Theseus based on the the corresponding target-template alignment. Structure superpositions of two PDB chains selected within the interface are generated using SALIGN (Braberg et al., 2012) from the Modeller Python library with default options and all feature weights set to 1.

## Implementation details

The server is built in Python (using the Django web framework), HTML and JavaScript (using ExtJS library). Apache Solr and Django Haystack are used for filtering the PDB chains and HHSearch hits by annotations. Django Celery, Celery and RabbitMQ are used for managing job tasks. The APE Server is used for Comet client-server communication. For manipulation of biological data, the server utilizes PyCogent (Knight et al., 2007) and to a lesser extent BioPython (Cock et al., 2009) and Modeller (Sali and Blundell, 1993) Python library.

Berman,H. et al. (2003) Announcing the worldwide Protein Data Bank. *Nature Structural & Molecular Biology*, **10**, 980–980.

Biasini,M. et al. (2010) OpenStructure: a flexible software framework for computational structural biology. *Bioinformatics*, **26**, 2626–2628.

Braberg,H. et al. (2012) SALIGN: A Webserver for alignment of multiple protein sequences and structures. *Bioinformatics (Oxford, England)*.

Cheng,J. et al. (2005) SCRATCH: a protein structure and structural feature prediction server. *Nucleic Acids Res.*, **33**, W72–76.

Cock,P.J.A. et al. (2009) Biopython: freely available Python tools for computational molecular biology and bioinformatics. *Bioinformatics*, **25**, 1422–1423.

Golovin,A. and Henrick,K. (2008) MSDmotif: exploring protein sites and motifs. *BMC Bioinformatics*, **9**, 312.

Jones,D.T. (1999) Protein secondary structure prediction based on position-specific scoring matrices. *J. Mol. Biol.*, **292**, 195–202.

Kabsch,W. and Sander,C. (1983) Dictionary of protein secondary structure: pattern recognition of hydrogen-bonded and geometrical features. *Biopolymers*, **22**, 2577–2637.

Knight,R. et al. (2007) PyCogent: a toolkit for making sense from sequence. *Genome Biol.*, **8**, R171.

Madej,T. et al. (2012) MMDB: 3D structures and macromolecular interactions. *Nucleic Acids Res.*, **40**, D461–464.

Sali,A and Blundell,T.L. (1993) Comparative protein modelling by satisfaction of spatial restraints. *J. Mol. Biol.*, **234**, 779–815.

Söding,J. (2005) Protein homology detection by HMM-HMM comparison. *Bioinformatics*, **21**, 951–960.

Spirin,S. et al. (2007) NPIDB: a database of nucleic acids-protein interactions. *Bioinformatics*, **23**, 3247–3248.

The UniProt Consortium (2011) Reorganizing the protein space at the Universal Protein Resource (UniProt). *Nucleic Acids Research*, **40**, D71–D75.

Ward,J.J. et al. (2004) Prediction and functional analysis of native disorder in proteins from the three kingdoms of life. *J. Mol. Biol.*, **337**, 635–645.
